# Dentists’ perception, knowledge, and clinical management of molar-incisor-hypomineralisation in Kuwait: a cross-sectional study

**DOI:** 10.1186/s12903-018-0498-2

**Published:** 2018-03-07

**Authors:** Abrar Alanzi, Anfal Faridoun, Katerina Kavvadia, Aghareed Ghanim

**Affiliations:** 1 Department of Developmental and Preventive Sciences, Faculty of Dentistry, Kuwait University, P.O. Box: 24923 – Safat, 13110 Kuwait City, Kuwait; 2 Ministry of Health, Kuwait City, Kuwait; 3 National and Kapodistrian University of Athens, Athens, Greece; 4 University of Melbourne, Melbourne, Australia

**Keywords:** Molar incisor hypomineralisation, Perception, Developmental defects, General dental practitioners

## Abstract

**Background:**

Molar-incisor Hypomineralisation (MIH) is considered as a global dental problem. There is little knowledge of general dental practitioners (GDPs) and dental specialists (DSs) about this condition in different parts of the world, particularly in Gulf Cooperation Council (GCC) countries. Hence, this study has been carried out to assess the knowledge of GDPS and DSs in Kuwait about MIH condition, its clinical presentation and management. Findings would help national school oral health program (SOHP) to promote good oral healthcare.

**Methods:**

A structured questionnaire was distributed to 310 attendees of the 18th Kuwait Dental Association Scientific Conference, Kuwait. Data concerning demographic variables, prevalence, diagnosis, severity, training demands and clinical management of MIH were collected.

**Results:**

A response rate of 71.3% (221/310) was reported. 94% of respondents noticed MIH in their practice. Yellow/brown demarcation has been observed as a common clinical presentation (> 50%). Almost 10–20% of MIH prevalence has been reported by the participants. Resin composite was the dental material often used in treating MIH teeth (~ 65%), and fewer than half would use it for treating moderately affected molars. Most respondents would use preformed metal crowns for severe MIH (63%). Dental journals were the information source for DSs; whereas, the internet was the information source for GDPs. Child’s behaviour was the main reported barrier for treatment of MIH affected children. Many GDPs felt unconfident when diagnosing MIH compared to dental specialists. Respondents supported the need to investigate MIH prevalence and to receive a clinical training.

**Conclusions:**

Molar incisor hypomineralisation is a recognised dental condition by practitioners in Kuwait. Yellow/brown demarcated opacities were the most reported clinical presentation, and the composite resin was the most preferred dental material for restoring MIH teeth. Most GDPs and dental specialists would use preformed metal crowns for severely affected molars. GDPs reported low levels of confidence in MIH diagnosis which necessitates conducting continuing education courses to provide high- quality dental care for children with MIH.

## Background

In 2001, Weerheijm et al. introduced the term Molar- Incisor Hypomineralisation (MIH) defining a specific clinical condition of a qualitative enamel developmental defect of systemic origin that affects one or more first permanent molars (FPMs) with or without the involvement of permanent incisors [1]. Other terms introduced before that were hypomineralised FPM [2], idiopathic enamel hypomineralisation in FPM [3], non-fluoride hypomineralisation [4] and cheese molars [5]. Reports exist on the prevalence of MIH lesions in all teeth and have shown that the second primary molars, which form at a similar time as the FPM, can also be affected with the condition defined as Hypomineralised Second Primary Molar (HSPM) [6, 7]. The global registered prevalence of MIH ranges from 2.4% to 40% and differs between countries [6, 8, 9]. There are limited numbers of MIH studies in Asia and the Middle East region.

Clinically, the presentation of MIH-affected teeth might be asymmetrical and varies from mild opacities to sever post-eruptive breakdown [9]. The severity of MIH may be different in the same patient, one to four first permanent molars may be affected. MIH can be difficult to diagnose, and clinicians may confuse it with other conditions such as enamel hypoplasia, fluorosis and amelogenesis imperfecta. Moreover, the diagnosis can be further complicated by the presence of carious lesions due to the rapid caries formation and progression [10].

In severe cases, MIH affected teeth are hypersensitive to thermal and mechanical stimuli, which might be a barrier to perform effective oral hygiene. Those teeth are at high risk of dental caries due to the rapid structural breakdown and inadequate oral hygiene. This would lead to a greater demand for extensive dental treatment and referral for specialists’ care [11]. Consequently, families can face financial issues to treat such teeth.

The detection and awareness of MIH are related to its recognition by dental practitioners. The first study that investigated the awareness of paediatric dentists in Europe with MIH condition was published in 2003 by Weerheijm et al. [12]. It showed that the majority considered MIH to be a clinical problem. Based on that study, similar investigations including general dental practitioners were carried out in Australia [13, 14], Iraq [15], Iran [16], Malaysia [17], and recently in Saudi Arabia [18] and UK [19]. Most of the dental practitioners from these countries reported that MIH affected teeth constitute a prevalent clinical problem and experienced difficulties in the diagnosis and clinical management.

Within the context of Kuwait, there is a lack of data on MIH prevalence in Kuwaiti children. However, caries prevalence in 12-year old school children has remained high (~ 26%) [20]. This high prevalence of dental caries may be partly attributed to some undiagnosed developmental enamel defects, such as MIH. In Kuwait, oral health care for school children in the public sector is mainly provided by general dental practitioners (GDPs) working in School Oral Health Program (SOHP). It is the only school-based oral health program in the Gulf region which covers a large student population. Since GDPs are not trained paediatric dentists, and they are in primary contact with children in SOHP dental clinics, it is not known if those GDPs are familiar with MIH condition or if sufficient information has been provided to them. Early diagnosis and referral for specialist care at the right time will aid in the appropriate management of children with MIH affected teeth.

Therefore, the purpose of this study is to assess the knowledge of general dental practitioners and dental specialists, who provide dental care for children in Kuwait, about MIH clinical condition considering its diagnosis, prevalence, severity and clinical management.

## Methods

The study population was general dental practitioners and dental specialists who were members of the Kuwait Dental Association (KDA) and provided oral health care for children. Participants were recruited during the 18th Kuwait Dental Association Scientific Conference held in November 2014. Three hundred and ten registered attendees met the criteria and included in the study: (1) dentists providing oral health care for children in SOHP, (2) paediatric dentists and (3) dental specialists who reported providing dental services for children. Based on the current data of the Kuwaiti dental labour force, the minimum required respondents were predicted to be 155, with an estimated margin of error of 5% and 80% sample power. Participation was anonymous and voluntary. The study approval was obtained from the ethical committee of Kuwait University Health Sciences Centre and the conference organising committee.

A structured questionnaire based on the study questions used in previous surveys [14–18] was used for data collection. Brief information about MIH and the study aims were provided on the cover page. The questionnaire was tested by a pilot study conducted amongst a group of recent Kuwait university dental graduates. The questionnaire consisted of three main sections and was not expected to take longer than 10 min to complete. The first section obtained the socio-demographic characteristics of the participants (e.g. age, gender, year of practice, work sector and place of dental degree). The second section included five coloured clinical photographs showing the clinical features of MIH affected FPMs and incisors. Participants were asked to study these photos and answer the related questions about the MIH perception, clinical appearance, prevalence in the community, dental management, aspects of continuing dental education and participants’ willingness for further training. In the third section, 3 cases of MIH were presented, and participants were asked to choose their treatment of choice accordingly. All clinical photographs used in the survey were obtained from the personal photograph collection of the author KK.

Data were entered into an Excel spreadsheet then analysed using Statistical Package for the Social Science version 20.0 software (SPSS Inc., Chicago, Ill., USA). Descriptive statistics (frequencies, percentage, mean) were determined. Chi-square test was used for nominal or ordinal variables. Post hoc test, based on adjusted standardised residuals, was run to confirm where the significant differences occurred between the groups. The generated outcomes have been analysed on the basis of *p*-value equal or less than 0.05.

## Results

Of the potential 310 participants invited to take part in the survey, 238 agreed to participate. Seventeen participants were excluded because ten of them handed incomplete questionnaires and seven were not practising dentistry in Kuwait. The completed questionnaires were 221, which resulted in a response rate of 71.3%. Table 1 shows the demographic characteristics of the participants. The sample included 115 (52%) general dental practitioners (GDPs) and 106 (47.9%) dental specialists (DSs). Of those DSs, there were 41(38.7%) paediatric dentists (PDs) and 65 (61.3%) dental specialists in other fields who cared for paediatric patients. DSs were distributed as follows: 16 (24.6%) orthodontics, 15 (23.1%) operative dentists, 19 (29.2%) endodontists, and 15 (23.1%) oral surgeons. From the questionnaire, 51.3% of the GDPs were between the ages of 31 to 40 years while 43.4% of the DSs were between the ages of 41–50 years. The majority of respondents (66.5%) work in the public sector. Fewer than half of DSs (41.8%) had obtained their postgraduate degree overseas (mainly in Asia).

**Table 1:** Demographic characteristics of the study participants

Characteristic	Total *N* = 221 *N* (%)	GDPs *N* = 115 *N* (%)	Paediatric Dentists *N* = 41 *N* (%)	Other Dental Specialists *N* = 65 *N* (%)
Age Group				
≤ 30	35 (15.8)	33 (28.7)	2 (4.9)	0
31–40	98 (44.3)	59 (51.3)	17 (41.5)	22 (33.8)
41–50	62 (28.1)	16 (13.9)	16 (39.0)	30 (46.2)
≥ 51	26 (11.8)	7 (6.1)	6 (14.6)	13 (20.0)
Years of Practice				
< 5	90 (40.7)	76 (66.1)	6 (14.6)	8 (12.3)
6–10	57 (25.8)	22 (19.1)	18 (43.9)	17 (26.2)
11–15	41 (18.6)	8 (7.0)	9 (22.0)	24 (36.9)
> 15	33 (14.9)	9 (7.8)	8 (19.5)	16 (24.6)
Work Sector				
Public sector	147 (66.5)	88 (76.5)	26 (63.4)	33 (50.8)
Private sector	62 (28.1)	25 (21.7)	10 (24.4)	27 (41.5)
Combined	12 (5.4)	2 (1.8)	5 (12.2)	5 (7.7)
Degree Level				
DDS/DMD/BDM	111 (50.2)	110 (95.7)	0	0
Specialty Only	20 (9.1)	5 (4.3)	9 (22.0)	7 (10.8)
Speciality + MSc /PhD	90 (40.7)	0	32 (78.0)	58 (89.2)
Place of speciality degree ^a^				
Middle East	29 (26.4)	–	9 (21.9)	20 (30.8)
Asia	46 (41.8)	–	17 (41.5)	29 (44.6)
Europe	26 (23.6)	–	13 (31.7)	13 (20.0)
USA	8 (7.3)	–	2 (4.9)	2 (3.1)
Australia	1 (0.9)	–	0	1 (1.5)

^a^ *N* = 110; include degree level (specialty only and specialty + MSc/PhD)

Knowledge and perception of the responding GDPs and DSs about MIH are illustrated in Table 2. Around half of the participants (47%) had noticed hypomineralised teeth on a monthly basis during their practice and nearly third of them (27%) had noticed such teeth on a weekly basis. The most frequent clinical presentation was yellow/brown demarcated opacities (56%) and the least reported was post eruptive enamel breakdown (5.4%). A large number of the GDPs (72%) were unconfident in diagnosing MIH compared to DSs (6.6%) (χ^2^ (4) = 104.8; *p* < 0.001). Approximately 39% of participants believed that the prevalence in the community is 10–20% with no significant difference between the groups. More than two-thirds of the participants (69%) observed MIH lesions at a low frequency in the second primary teeth.

**Table 2:** MIH perception, clinical appearance and prevalence according to study participants

Question	GDPs *N* = 115 *N* (%)		Paediatric Dentists *N* = 41 *N* (%)		Other Dental Specialists *N* = 65 *N* (%)		*X* ^2^	*P* value
How often do you notice hypomineralised teeth in your practice?								
Never	7	(6.1)	2	(4.9)	5	(7.7)	4.75	0.576
Weekly basis	28	(24.3)	13	(31.7)	19	(29.2)		
Monthly basis	51	(44.4)	20	(48.8)	32	(49.2)		
Yearly basis	29	(25.2)	6	(14.6)	9	(13.9)		
Most frequently notice in your practice?								
White demarcated opacities	42	(36.5)	11	(26.8)	17	(26.2)	2.939	0.816
Yellow/brown demarcated opacities	60	(52.2)	26	(63.4)	38	(58.5)		
Posteruptive enamel breakdown	6	(5.2)	2	(4.9)	4	(6.1)		
Never seen	7	(6.1)	2	(4.9)	6	(9.2)		
How confident in diagnosing MIH teeth?								
Very confident	11	(9.6)	7	(17.1)	3	(4.6)	104.8	0.000*
Confident	21	(18.2)	32	(78.0)	57	(87.7)		
Unconfident	83	(72.2) ^a^	2	(4.9) ^b^	5	(7.7) ^b^		
Are you aware that MIH differs from fluorosis and hypoplasia?								
Yes	93	(80.9) ^b^	41	(100) ^a^	55	(84.6) ^b^	8.99	0.011*
No	22	(19.1)	0		10	(15.4)		
Prevalence of MIH might be in your community?								
< 5%	18	(15.7)	2	(4.9)	6	(11.8)	5.587	0.061
5–10%	36	(31.3)	10	(24.4)	10	(25.3)		
10–20%	38	(33.0)	19	(46.3)	29	(38.9)		
> 20%	7	(6.1)	2	(4.9)	10	(8.6)		
Not sure	16	(13.9)	8	(19.5)	10	(15.4)		
Would be worthwhile to investigate MIH prevalence?								
Yes	101	(87.8)	39	(95.1)	62	(95.4)	3.90	0.142
No	14	(12.2)	2	(4.9)	3	(4.6)		
How frequently do you notice this defect in the second primary molar?								
More frequently	11	(9.6)	0		4	(6.2)	21.49	0.001*
Less frequently	72	(62.6) ^b^	39	(95.1) ^a^	42	(64.6) ^b^		
Same as FPM	7	(6.1)	2	(4.9)	1	(1.5)		
Do not examine primary molars	25	(21.7)	0		18	(27.7)		

* *p* < 0.05 = significant difference

^a-b^ values within rows with different superscript letters are significantly different (*P* < 0.05) using post hoc test

The management considerations of MIH reported by the respondents and their source of MIH information are presented in Table 3. Resin composite was the most preferred dental material (64.7%), followed by glass ionomer cement (GIC, 35.7%) and preformed metal crowns (PMC, 32.1%). Other dental specialists use PMC less frequently (9.2%) when compared to GDPs (39.1%) and PDs (48.8%), (χ^2^ (2) = 23.4; *p* < 0.001). Regarding the reported barriers for treating children with MIH, many GDPs (60.9%) and almost half of the PDs (48.8%) considered child’s behaviour as an important barrier to provide proper treatment. For GDPs, long treatment time (38.3%) and insufficient training to treat MIH patients (31.3%) were other common barriers. Difficulty in achieving local anaesthesia was the second most common barrier (22%) among paediatric dentists. Other DSs were fairly evenly distributed across the three barriers: long treatment time (21.5%), insufficient training to treat children with MIH (18.5%), and child’s behaviour (16.9%). There was a significant difference between GDPs and all DSs in relation to “long treatment time” barrier (χ^2^ (2) = 10.6, *p* = 0.005), “child’s behavior” (χ^2^ (2) = 32.5, *p* < 0.001), and “insufficient training to treat MIH” barriers (χ^2^ (2) =17.9, p < 0.001). Regarding the information source of MIH knowledge, the internet was the main information source for GDPs (37.4%), while dental journals were the main source for all dental specialists (PDs = 63.4%, Other DSs = 55.8%). No significant difference was found between the groups regarding the information source (*p* = 0.103). The necessity of having a clinical training regarding tooth hypomineralisation has been strongly agreed by respondents (87%).

**Table 3:** MIH management considerations, source of information, and clinical training demand according to study participants

Question	GDPs *N* = 115 *N* (%)		Paediatric Dentists *N* = 41 *N* (%)		Other Dental Specialists *N* = 65 *N* (%)		*X* ^*2*^	*P* value
Type of dental materials often use in treating MIH tooth?								
Amalgam	10	(8.7)	9	(22.0)	11	(16.9)	5.407	0.067
Resin composite	75	(65.2)	24	(58.5)	44	(64.7)	0.95	0.622
GIC	47	(40.9)	14	(34.1)	18	(35.7)	3.196	0.202
PMCs	45	(39.1) ^b^	20	(48.8) ^b^	6	(9.2) ^a^	23.42	0.000*
Compomer	2	(2.6)	4	(9.8)	4	(6.2)	3.53	0.171
Barrier in performing MIH management								
Long treatment time	44	(38.3) ^a^	6	(14.6) ^b^	14	(21.5) ^b^	10.66	.005*
Child’s behaviour	70	(60.9) ^a^	20	(48.8) ^b^	11	(16.9) ^b^	32.51	.000*
Difficulty in achieving local anesthesia	27	(23.5)	9	(22.0)	8	(12.3)	3.381	0.184
Insufficient training to treat children with MIH	36	(31.3) ^a^	0		12	(18.5) ^b^	17.99	.000*
Are you receiving any information on MIH?								
Yes	75	(65.2)	34	(82.9)	44	(67.7)	4.552	0.103
Dental journals	29	(25.2)	26	(63.4)	33	(55.8)		
Continuing education	25	(21.7)	15	(36.6)	12	(18.5)		
Brochures or pamphlets	4	(3.5)	2	(4.9)	5	(7.7)		
Internet	43	(37.4)	23	(56.1)	29	(44.6)		
Books	27	(23.5)	8	(19.5)	12	(18.5)		
Others	7	(6.1)	0		0			
Need for clinical training regarding tooth hypomineralisation								
Diagnosis	21	(18.3)	3	(7.3)	8	(12.3)	2.153	0.341
Aetiology	11	(9.6)	6	(14.6)	7	(10.8)		
Treatment	33	(28.7)	8	(19.5)	16	(24.6)		
All	39	(33.9)	17	(41.5)	24	(36.9)		

* *p* < 0.05 = significant difference

^a-b^ values within rows with different superscript letters are significantly different (P < 0.05) using post hoc test

n and % in the table represent those of YES answers only

**Fig. 1:**
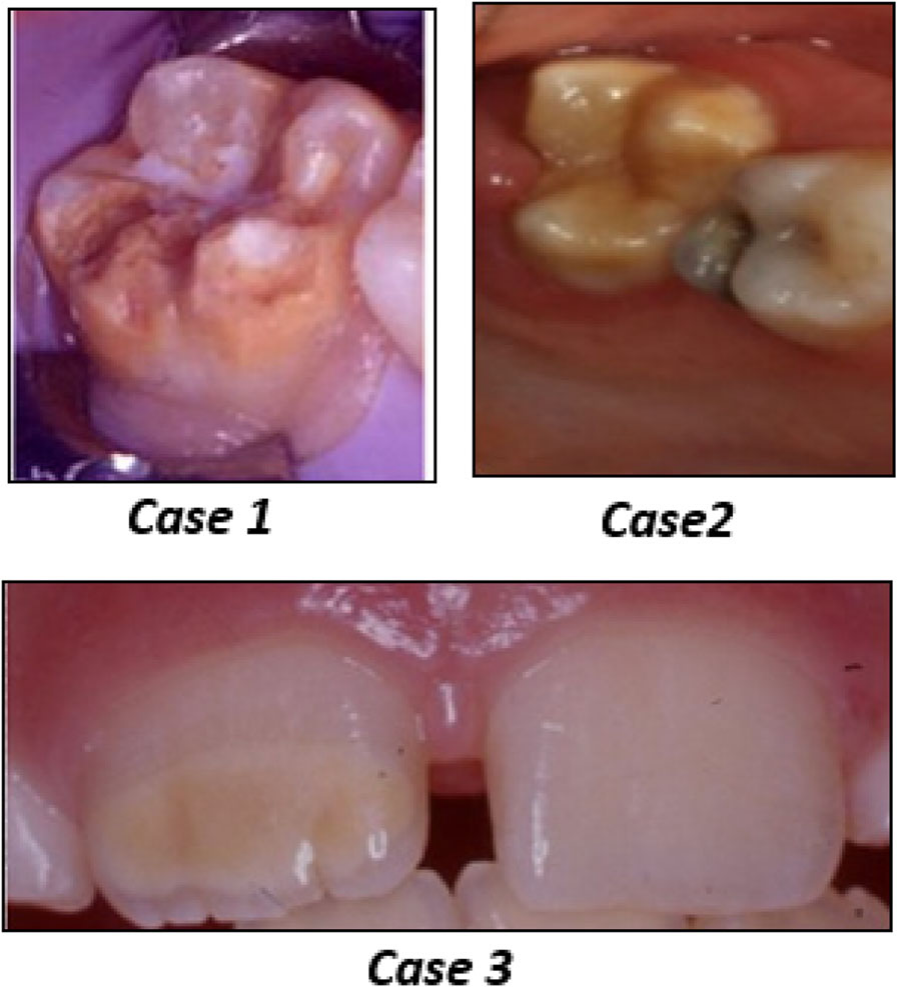
Clinical Photographs of the cases presented in the survey

Figure 1 presents three clinical cases that were used to test the participants’ knowledge regarding MIH management. As presented in Table 4, for severely MIH affected molar with post eruptive breakdown (Case 1), the vast majority of PDs (83%) and GDPs (64%) would use PMC compared to 50% of other DSs. Significant difference existed between the groups (χ^2^ (8) = 17.24; *p* < 0.05). Extraction was the least preferable treatment option by all participants (2.7%). In Case 2, about half of the GDPs (48%) and PDs (46%) would use composite resin restoration for moderately MIH affected molar whereas less than one-third of DSs (28%) would use composite resin (χ^2^ (10) = 28.29; *P* < 0.05). In Case 3, for mild MIH central incisor, bleaching and seal with low viscosity resin were the treatment of choice expressed by half of the dental specialists including paediatric dentists. Fewer than half of GDPs (43%) would remove the MIH affected area and restore with composite resin with no significant difference between the groups about this treatment option.

**Table 4:** Clinical case scenarios regarding MIH management and the responses of the study participants

Question	GDPs *N* = 115 *N* (%)		Paediatric Dentists *N* = 41 *N* (%)		Other Dental Specialists *N* = 65 *N* (%)		*X* ^*2*^	*P* value
CASE 1: 7 year old child with severely MIH affected tooth #16 and post eruptive breakdown								
PMCs	74	(64.3) ^b^	34	(82.9) ^b^	32	(49.2) ^a^	17.242	0.028*
Composite restoration & fissure sealant	23	(20.0) ^b^	2	(5.0) ^a^	19	(29.2) ^b^		
GI restoration	11	(9.6)	3	(7.3)	5	(7.7)		
Extraction	3	(2.6)	1	(2.4)	2	(3.1)		
Not sure what to do	4	(3.5)	1	(2.4)	7	(10.8)		
CASE 2: 6 year old child with moderate MIH on tooth #16								
PMCs	23	(20.0) ^b^	10	(24.4) ^b^	4	(6.2) ^a^	28.294	.002*
Composite restoration	55	(47.8) ^b^	19	(46.3) ^b^	18	(27.7) ^a^		
Fissure sealant	13	(11.3)	1	(2.4)	14	(21.5)		
GI restoration	20	(14.4)	9	(22.0)	21	(32.3)		
Extraction	1	(0.9)	0	(0.0)	1	(1.5)		
Not sure what to do	3	(2.6)	2	(4.9)	7	(10.8)		
CASE 3: 9 year old child with mild MIH affecting tooth #11								
Microabrasion	29	(25.2)	9	(22.0)	11	(16.9)	13.646	0.034*
Etch, bleach, and seal with low viscosity resin (ICON ®)	29	(25.2) ^a^	19	(46.3) ^b^	30	(46.2) ^b^		
Remove MIH affected area and restore with resin	49	(42.6)	11	(26.8)	17	(26.2)		
Not sure what to do	8	(7)	2	(4.9)	7	(10.8)		

* *p* < 0.05 = significant difference

^a-b^ values within rows with different superscript letters are significantly different (P < 0.05) using post hoc test

## Discussion

Molar incisor hypomineralisation has become more apparent clinical condition and a field of interest to dental practitioners worldwide. Little is known about MIH condition in the Middle East region, especially in Kuwait as part of Gulf Cooperation Council (GCC) countries. Exploring the awareness and knowledge of clinicians on MIH topic is the foundation to plan strategies for delivering high quality and efficient oral health care to children. The present study is the first published study to investigate the awareness, knowledge, and clinical experiences of MIH condition among general dental practitioners and dental specialists in Kuwait. The study findings highlight the importance of recognising MIH condition and advocate for future collaborative efforts between GCC countries to explore its prevalence and aetiology in this part of the world.

The majority of participants had encountered the presence of MIH teeth in their clinical practice, consistent with the results of previous studies in the region [15, 16, 18]. More than one-third of the respondents believed that the prevalence in Kuwait would be between 10 to 20%. This perceived prevalence was in line with the documented prevalence in other neighbouring countries [21–23]. There is a high need for a future epidemiological study to determine the actual prevalence of MIH in Kuwaiti children. Yellow/brown demarcated opacities were perceived by the participants like the most frequent enamel defect in line with previous reports [15–18]. The prevalence of post-eruptive enamel breakdown was low in the surveyed population. This finding might be masked by extensive caries or atypical restoration as reported by previous research [11].

Many GDPs in this study were unconfident about diagnosing MIH when compared to dental specialists, which explains their request for a further clinical training course on MIH condition. A similar request for a training course regarding MIH-aetiological and therapeutic fields was reported among Saudi dentists [18]. Early detection of MIH teeth is essential to facilitate the curative treatment due to the rapid breakdown nature of MIH affected areas [11]. Most of the respondents observed MIH condition at a low frequency in second primary molar teeth, which is described as “Hypomineralised Second Primary Molars” (HSPM). The presence of HSPM can be considered a predictor for MIH and requires following up with these patients more frequently [7]. However, the absence of HSPM does not exclude the future development of MIH in the succedaneous teeth [7, 24].

In the present study, the most preferred dental material used by respondents was composite resin. The findings were consistent with other studies [16, 17], but not in agreement with the results of Crombie et al. [14] which found GIC to be the preferred material. The majority of paediatric dentists use PMC more frequently to manage severely MIH-affected molars compared to other dental specialists, who rarely consider it as a treatment option. This could be attributed to the proper training of paediatric dentists during their residency programs in preparing and placing PMC. A considerable number of general practitioners in this study also would use PMC which was similar to the number of Australian professionals in a previous study [13]. The current survey had identified “child’s behaviour” to be the common barrier for MIH management that may reflect the inadequate training of general practitioners in child’s management. For paediatric dentists, it is likely that difficult child behaviour might be a result of other factors affecting the management of MIH such as inadequate control of pain and teeth sensitivity [10]. The finding is distinctive from the results of Hussein et al. [17], who found “insufficient training” on how to treat MIH as a common barrier among the dentists in Malaysia.

The three presented clinical cases in this study showed a general agreement on using PMCs for severe MIH affected molars by the respondents. Although around a third of other dental specialists would utilise composite resin in such situation, placement of PMC is still recommended for teeth with multiple defects to provide full-coverage and long-term retention [25]. However, PMC can be detrimental to the periodontal health that disfavours its use as a permanent restoration [26]. Recently, a new interim treatment alternative was described for a molar with severe MIH using a glass ionomer restoration followed by a placement of an orthodontic band. This interim technique showed a successful result after 18 months of follow-up without further intervention [27]. Extraction was the least selected treatment option for severely affected molar presented in the case. This reflects a more conservative approach of tooth preservation rather than scarifying the whole tooth. However, the extraction of first permanent molars severely affected by MIH can be considered as a suitable and cost-effective treatment alternative in some clinical situations. Recent research showed a favourable space closure without orthodontic intervention if the extraction of defective FPM is performed at the optimal age and prior to the eruption of second molars [28, 29]. The decision to extract any of the FPM should be evaluated and discussed with an orthodontist if a good outcome is expected [30].

For moderately MIH-affected molars, composite resin was the treatment of choice for both GDPs and PDs while many other DSs chose glass ionomer cement. Restorations with GIC or RMGIC materials are not recommended in the stress-bearing areas of permanent molar teeth and could be used as interim restorative materials until placing a definitive coronal restoration [31]. Lygidakis et al. [30] reported a successful result of composite resin restoration after four years of placement on two or more surfaces of affected MIH molars. On the other hand, Mejare et al. [32] and Kotsanos et al. [33] found a considerable failure rate with composite resin and a need for additional retreatment. Further long-term clinical trials with a defined treatment procedure and methodology criteria are required to reveal the type of composite material most appropriate for MIH affected teeth.

It was shown that approximately 72% of children who have MIH affected molars tend to have affected incisors as well [34, 35]. The most frequently found association of affected MIH was 4 M/2 incisors (23.5%) with all 12 index teeth erupted [35]. Also, it has been reported that 95.1% of MIH affected incisors were mild. In the third clinical case involving MIH affected incisor, half of GDPs would remove the affected area and restore it with direct composite resin. Whereas, half of paediatric dentists and other dental specialists would use the etch-bleach-seal technique, which has been advocated by Wright [36]. Restoration with direct composite resin can be an alternative choice for larger enamel defects [37].

Despite the fact that this type of study has validity problems in which respondents can over or under report specific details, the findings of the current survey establish a baseline data about MIH in Kuwait. In addition, it would be the foundation for Kuwait SOHP to promote good oral healthcare by running evidence-based training courses on MIH condition in order to deliver an appropriate care for children with MIH. Further to this, there is a need for determining MIH prevalence among the children in Kuwait as well as investigating its distribution and severity.

## Conclusion

The current study shows that molar incisor hypomineralisation is a condition commonly encountered by general dental practitioners and dental specialists in Kuwait. Yellow/brown demarcated opacities were perceived by the respondents like the most common clinical presentation of MIH. The majority of GDPs felt unconfident in diagnosing MIH compared to dental specialists. Child’s behaviour was the most frequently reported barrier to MIH management. A general agreement between GDPs’ and dental specialists’ views was found on the use of PMCs for treating severely affected molars. Composite resin was preferred by both GDPs and paediatric dentists for treating moderately affected molar. A variation in views was recorded about the proper treatment of MIH affected incisors. Continuing education courses on MIH condition are required to ensure high-quality care for children with MIH affected teeth.
